# Application of back propagation neural network in complex diagnostics and forecasting loss of life of cellulose paper insulation in oil-immersed transformers

**DOI:** 10.1038/s41598-024-56598-x

**Published:** 2024-03-13

**Authors:** M. K. Ngwenyama, M. N. Gitau

**Affiliations:** Department of Electrical, Electronic and Computer Engineering, University of Pretoria, Pretoria, 0002 South Africa

**Keywords:** 2-Furaldehlyne (2FAL), Back propagation neural network (BPNN), Degree of polymerization (DP), Loss of life (LOL), Transformer health index (HI), Engineering, Electrical and electronic engineering

## Abstract

Oil-immersed transformers are expensive equipment in the electrical system, and their failure would lead to widespread blackouts and catastrophic economic losses. In this work, an elaborate diagnostic approach is proposed to evaluate twenty-six different transformers in-service to determine their operative status as per the IEC 60599:2022 standard and CIGRE brochure. The approach integrates dissolved gas analysis (DGA), transformer oil integrity analysis, visual inspections, and two Back Propagation Neural Network (BPNN) algorithms to predict the loss of life (LOL) of the transformers through condition monitoring of the cellulose paper. The first BPNN algorithm proposed is based on forecasting the degree of polymerization (DP) using 2-Furaldehyde (2FAL) concentration measured from oil samples using DGA, and the second BPNN algorithm proposed is based on forecasting transformer LOL using the 2FAL and DP data obtained from the first BPNN algorithm. The first algorithm produced a correlation coefficient of 0.970 when the DP was predicted using the 2FAL measured in oil and the second algorithm produced a correlation coefficient of 0.999 when the LOL was predicted using the 2FAL and DP output data obtained from the first algorithm. The results show that the BPNN can be utilized to forecast the DP and LOL of transformers in-service. Lastly, the results are used for hazard analysis and lifespan prediction based on the health index (HI) for each transformer to predict the expected years of service.

## Introduction

An electrical transformer is a critical component of electricity transmission and distribution networks. An oil-immersed transformer is the most expensive equipment in the electrical system, and its failure would lead to widespread blackouts and catastrophic economic losses^1,2^. Therefore, it is vital to perform routine maintenance and continuous monitoring of electrical transformers, particularly those employed at critical locations. Several massive electrical transformers that have been in service in recent years have been quite ancient^3^. These very ancient units are still in use, owing predominately to economic constraints. Presently, mankind has become increasingly reliant on the provision of power, imposing strain on the stability, availability, and cost-effectiveness of power supply^4^. The trouble-free operation of electrical transformers is a crucial condition for electrical system reliability and security. However, among the primary reasons for the failure of aging transformers in^5,6^, the mechanical integrity of the units to withstand stress caused by short-circuit currents can deteriorate dramatically with the degrading of insulation material. As a result, it is critical to thoroughly assess the aging state of the insulation material. The withering and lifespan duration of an oil-immersed transformer is determined by the solid insulation and the level of the withering of the insulating paper^7–10^. Oil has a strong insulating property while also operating as a cooling medium via natural or induced circulation. The oil-impregnated paper acts as an electrical insulator between windings and a mechanical barricade between individual windings and winding layers. As a result, the paper serves a major vocation in paper-oil insulation. Poor paper integrity causes early insulation deterioration, which potentially contributes to transformer collapse following a winding short circuit, for instance^11,12^.

Presently, the approaches for evaluating electrical transformers are separated into two categories: online supervision and offline supervision. With the evolution of the units, online system supervision has become more prevalent. While the transformers are in operation, online supervision approaches may be utilized to inspect and assess operational efficiency, evaluation, emergency warning action, and establish effective maintenance and repair forecasts^13,14^. Most significantly, using analysis, these online supervision systems can estimate the residual expected lifespan. However, monitoring approaches in this discipline are relatively recent. Research and innovation studies are constantly improving, although, it is still a challenge to determine the residual life of an electrical transformer since residual-life calculations are dependent on a variety of conditions. According to present requirements, the structural lifespan of an electrical transformer is restricted to the duration of the insulation paper. Deterioration of an insulation paper can be diagnosed using the degree of polymerization (DP), Furan analysis (FA), and ${\text{CO}}_{2}\text{/CO}$ ratio analyses^15,16^. DP analyses measure the physical capability of paper by calculating the cellulose degree. However, DP analyses and other tests fail to predict the operational lifespan of the transformer since numerous other factors influence the insulation degradation process^17,18^. Accurate detection and mining of certain dissolved gases in dielectric transformer oil has become the fastest-growing procedure in the diagnosis of transformer faults. Insulation breakdown occurs over time and is affected by heat, humidity, and oxygen concentration. Sophisticated oil conservation systems can reduce humidity and oxygen impacts on insulation breakdown^19,20^. The main determining factor in withering is the insulation temperature. Consequently, in practice, the extent of the cellulose paper withering is influenced by the transformer’s hotspot temperature. Tensile strength and DP are measured to determine the mechanical qualities of cellulose paper^21^. These characteristics are employed to assess when the cellulose paper insulation reaches the end of its dependable life. The cellulose paper insulation end-of-life requirements are commonly proposed^22^ to have DP values of 150–250; below 150, the cellulose paper is considered to be mechanically weak. The assessment of paper insulation for its DP value requires the extraction of a few sheet strips of paper from the investigated transformers^23,24^. However, the process remains challenging. Oil samples are utilized as an alternative. The procedure can conveniently be carried out during transformer maintenance, service, or repairs since it is usually not practical (and often dangerous to the transformer) to obtain the cellulose paper sample from a de-energized, in-service transformer. It has been demonstrated in^25–28^, that the amount of 2-Furaldehlyne (2FAL) present in oil (the most important component of cellulose paper degradation) is significantly related to the DP of the cellulose paper within the transformer. The levels of 2FAL in oil correspond to the typical degradation of the cellulose paper. By measuring the quantity and types of furans present in a transformer oil sample, the cellulose paper insulation overall DP can be inferred with a high degree of confidence. The types and concentration of furans in an oil sample can also indicate abnormal stress in a transformer, whether intense, short-duration overheating or prolonged, general overheating. Therefore, estimating their residual lifespan is critical to prevent transformers from being shut down prematurely during service^29,30^. Table 1 illustrates the furan concentration thresholds used to grade the transformer condition. The value of DP and the amount of furan can be used to determine the condition of cellulose paper.

**Table 1:** Furan concentration in parts per million (ppm)^31^.

Furan concentration (ppm)	DP value	Significance
0–0.1	1200–700	Healthy transformer
0.1–1.0	700–450	Moderate degradation
1–10	450–250	Extensive degradation
> 10	< 250	End-of-life criteria

The furan test can simply determine furan from oil sampling on an in-service transformer. As demonstrated in Table 2, the quantity of furan concentration contained in the cellulose paper is used to calculate the transformers’ service life in years.

**Table 2:** The age profile of cellulose paper based on furan^32^.

Furan concentration (ppm)	Service life (years)
0–0.1	< 20
0.1–0.25	20–40
0.25–0.5	40–60
0.5–1.0	> 60
> 10	–

Several DP and loss of life (LOL) techniques for electrical transformers have been proposed recently, in^33,34^, however, the current standards and approaches possess limitations, such as inaccurate estimations and inconsistent results for similar oil samples. These challenges must be rectified to have an efficient lifespan prediction scheme. Computational techniques have also been utilized to address these challenges. DGA has been conducted utilizing wavelet networks (WN)^35,36^, expert systems (ES)^37^, adaptive neuro-fuzzy inference system (ANFIS)^38^, artificial immune networks (AIN)^39^, support vector machines (SVM)^40^, and fuzzy logic (FL)^41^. Although the results for LOL of transformers presented in this work are decent, it is essential to promote the proposed approach to enhance LOL prediction accuracy to provide dielectric testing facilities and transformer manufacturers with reliable alternatives to existing techniques. Table 3 presents a comparative study of the recent research works and the proposed algorithm for forecasting LOL of cellulose paper insulation.

**Table 3:** Summary of recent research works and proposed algorithm.

Refs.	Years	Proposed technique	Contribution
^42^	2021	Regression modeling	The study estimated DP utilizing the furfural marker at different oil-to-pressboard ratios and oil change statuses
^43^	2022	Artificial Neural Network (ANN)	The study estimated furans by analyzing temperature, carbon dioxide, carbon monoxide, and moisture to estimate DP
^44^	2022	Empirical modeling	The study estimated DP utilizing methanol $(\text{MeOH)}$ concentrations obtained at low temperatures. The relative error was 7%
^45^	2023	ANFIS, Roger’s ratio approach	A hybrid Rogers ratio technique-based ANFIS was proposed to detect transformer faults. The training was carried out by employing the gas ratios presented by the IEEE C57-104 and IEC 60599 standards
^46^	2024	Multi-classification model	The study analyzes DGA by using machine learning (ML) techniques, adherence to IEC 60599:2022, and Eskom (Specification—Ref: 240-75,661,431) standards
Current study	2024	Back Propagation Neural Network (BPNN)	Presented in section “Introduction” (Paper contribution)

### Paper contribution

**Figure 1:**
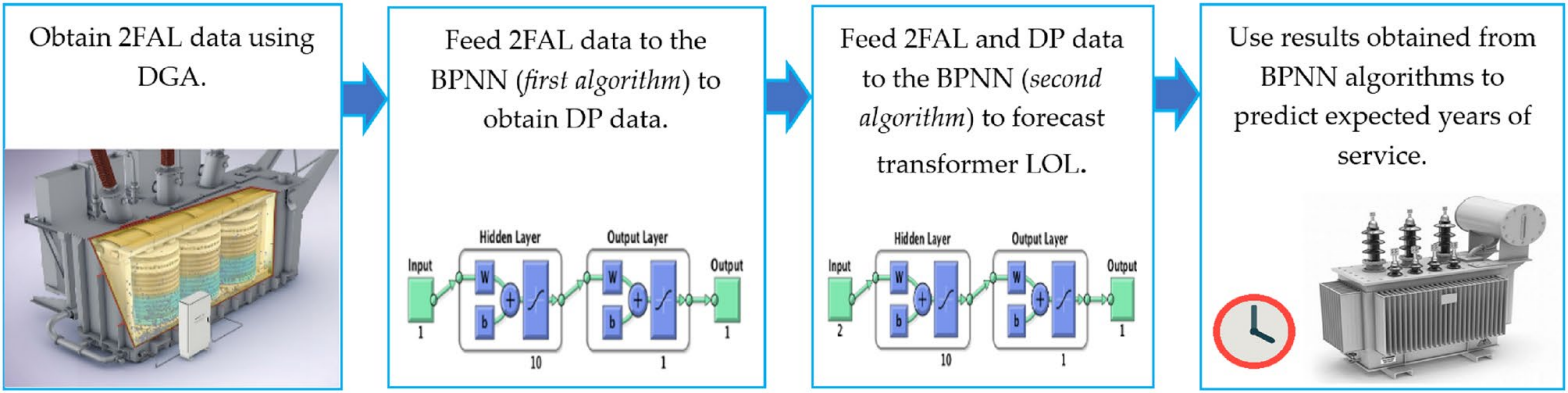
Contribution stages block presented in a block diagram.

In this work, an elaborate diagnostic approach is proposed to evaluate twenty-six different transformers in-service to determine their operative status. The solid insulation evaluation process is a reliable practice to assess and forecast the DP and LOL as it provides generous information in inspecting the transformer condition. The contributions of the current research study are as follows and demonstrated in a block diagram as shown in Fig. 1.

- DGA is utilized to obtain 2FAL data from oil samples received from all transformers under investigation.
- A BPNN algorithm is proposed to forecast the DP using 2FAL concentration measured from oil samples using DGA.
- A second BPNN algorithm is proposed to forecast the transformer LOL using the 2FAL and DP data obtained from the first BPNN algorithm.
- Lastly, the results are used for hazard analysis and lifespan prediction based on the health index (HI) for each transformer to forecast the expected years of service as per the IEC 60599:2022 standard^47^ and CIGRE brochure^48^.

According to current research works, ${\text{MeOH}}$ is commonly used to analyze cellulose paper in oil transformers. The experimental investigations reported and addressed in^49–52^ have revealed that ${\text{MeOH}}$ is a potential degradation indicator for all cellulose-based materials and may be used in many types of mineral oil. There is a significant relationship between DP and ${\text{MeOH}}$ formation, as well as earlier diagnosis of cellulose degradation than 2FAL concentrations, supporting published works. However, at high temperatures, ${\text{MeOH}}$ concentrations tend to stabilize while only 2FAL concentrations keep on developing^53^. Nonetheless, it is critical to recognize that early detection $(\text{MeOH)}$ is critical for effective monitoring and mitigation measures at the appropriate time. In this work, the fact that 2FAL compounds are constantly produced, even at a later stage than ${\text{MeOH}}$, lends further weight to employing 2FAL data to forecast the transformer LOL utilizing the proposed BPNN algorithms since they can learn and train continuously growing 2FAL concentrations without experiencing overfitting. The results obtained using the proposed approach will be used to decide whether to repair, monitor, service, or scrap the transformer under investigation^10,54^.

The currently available capabilities and limitations of the proposed BPNN algorithm in comparison to other types of learning algorithms are presented in Table 4. The performances of the learning algorithms are evaluated according to how well they can generalize the relationship between vibration parameters, damage locations, and severities across a range of input and output variable counts^55^.

**Table 4:** Capabilities and limitations of the BPNN in comparison to other learning algorithms that are currently available.

	BPNN [current study]	RBFN^56^	PNN^57^	CPNN^58^
Application scope	Classification Regression	Classification Regression	Classification	Classification
Classification process	Minimize sum squared errors by updating weights	Relies upon the BPNN process	Bayesian decision rule	Uses clustering methods and PNN process
Capabilities	Simple application to predict the patterns Does not require any statistical features in the learning processes Easy to identify the magnitude of attributes based on weights A variety of applications are available	Simpler format of Gaussian function Radial basis function nodes can be substituted with different functional forms Performs relatively well in both smaller and larger datasets	Simple architecture (no backpropagation) Relatively good accuracy in classification problems Insensitive to noise points	A smaller network size than ordinary PNN can avoid saturation of the Parzen window which leads to misclassification May be more applicable because it provides knowledge of the relative importance between explanatory variables Fast training time
Limitations	Easily get stuck in local minima, resulting in a suboptimal solution Prone to overfitting and underfitting	Constructing network architecture is complicated Long training time No sufficient observations	More computationally than BPNN (pre-stored pattern neurons) Saturated Gaussian can lead to misclassification	May not provide higher prediction accuracy than PNN for discrete choice data Varied by several clusters determined in *k*-means clustering

For future work, the authors will evaluate ${\text{MeOH}}$ and 2FAL concentrations using artificial intelligence algorithms. The conclusions about which data produces accurate results using the algorithms presented in Table 3 will be drawn from which data produces underfitting and overfitting during training^59^.

### Paper organization

The rest of the work is structured as follows: Section “Methods used to investigate transformers” outlines the methods used to investigate transformers in-service. Section “Results and analysis” provides a discussion of the results and analysis of the investigated transformers. Section “Discussion” provides a discussion and recommendations, and the paper is concluded in section “Conclusion”.

## Methods used to investigate transformers in-service

**Figure 2:**
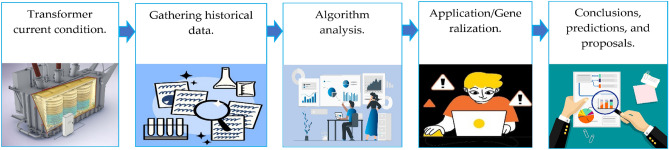
Phases for technical diagnosis.

The practical diagnostic approach for estimating the current status and remaining operating lifespan of an electrical transformer in terms of expected End of Life (EOL)^60^ is discussed in this Section. Four different tactics for analyzing data from on-site testing and estimating using algorithms were combined to develop the diagnostic technique. Oil tests were combined for estimating dissolved gases and oil quality, DP calculations based on indirect measurements of dissolved 2FAL in transformer oil, the HI approach for processing data collected through visual inspection, history, and test results, and risk of failure calculation based on the HI^61,62^. These techniques were utilized to determine whether transformers should be repaired, closely monitored, maintained, or scrapped^63,64^. Twenty-six specific transformers utilized in distribution substations in South Africa, Mpumalanga, were exposed to the proposed diagnostics. The diagnostics were divided into five phases, which are described below^25,65–68^:

- Assessment of the transformer’s current condition: involves visual inspection and all DGA and chemical tests conducted on transformer oil.
- Gathering historical data: involves gathering information from prior faults, repairs, and maintenance. Obtaining data for the transformer’s load history and analyzing yearly test reports.
- Algorithm analysis: the outcomes of each test are evaluated and applied to various algorithms to gather more data on the technical status of the transformers, as well as to detect hidden issues and abnormalities.
- Application/Generalization: all data obtained through various tests and algorithms is generalized and analyzed to obtain more detailed knowledge about the transformer’s present condition. The majority of the tests usually yield comprehensive data for individual transformer parts.
- Conclusions, predictions, and proposals: conclusions are reached and predictions are provided regarding the likelihood of failure, EOL of the transformers, and dependability according to the outcomes of diagnostic tests and procedures used. Proposals are provided to prioritize maintenance for the most degraded transformers or to discard them if the end of life has passed. Figure 2 demonstrates a block diagram of the five phases utilized in technical diagnosis.

**Figure 3:**
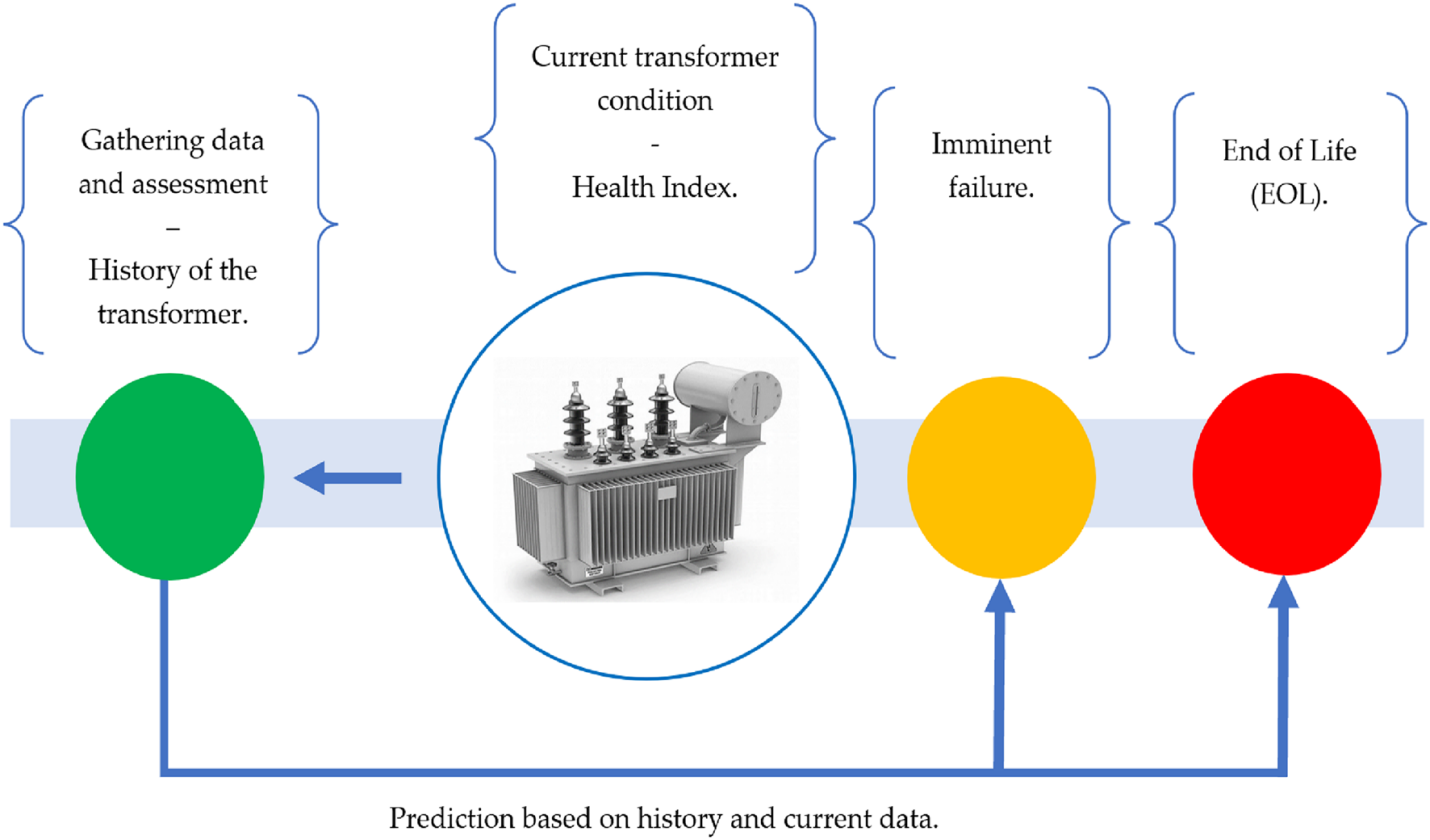
Predicting imminent failures and EOL based on HI and historical data.

The data gathered from the transformer’s historical events, conducted tests, as well as visual inspection is utilized to forecast imminent failures and EOL of each transformer, and according to these factors, a decision is made to prioritize transformers for repair, maintenance, or replacement if a transformer has reached its EOL^69^. Figure 3 demonstrates the primary descriptive framework of the approach utilized, which relies on HI calculations^70,71^.

The inspected transformers were manufactured throughout a variety of periods, and some of them had been in service for more than 30 years. The rated power ranged from 1250 kVA to 3.2 MVA, and the primary and secondary voltages were 11 kV, 6.6 kV, and 400 V, respectively. Transformer designs changed based on the year they were manufactured. Several transformers had undergone maintenance over their existence. The cooling system designs of transformers ranged from Oil Natural Air Natural (ONAN); Oil Natural Air Forced (ONAF); Oil Forced Air Forced (OFAF); Oil Natural Water Forced (ONWF); and Oil Forced Water Forced (OFWF)^72,73^. Table 5 contains technical information on the transformer group under consideration^72^.

**Table 5:** Technical data of inspected transformers.

Index	Serial number	Rated power and voltage	Manufactured year
T1	30,661,407/01	1250 kVA, 11 kV/400 V	1985
T2	30,661,409/01	1250 kVA, 11 kV/400 V	1987
T3	30,661,410/01	1250 kVA, 11 kV/400 V	1988
T4	30,661,404/01	1250 kVA, 11 kV/400 V	1984
T5	30,661,408/01	1250 kVA, 11 kV/400 V	2000
T6	30,661,411/01	1250 kVA, 11 kV/400 V	1984
T7	30,661,415/01	1250 kVA, 11 kV/400 V	2003
T8	30,661,405/01	2500 kVA, 6.6 kV/11 kV	1986
T9	30,661,413/01	2500 kVA, 6.6 kV/11 kV	2000
T10	30,661,401/01	2500 kVA, 6.6 kV/11 kV	2002
T11	30,661,402/01	2500 kVA, 6.6 kV/11 kV	1985
T12	30,661,418/01	2500 kVA, 6.6 kV/11 kV	1987
T13	30,661,403/01	3.2 MVA, 11 kV, 400 V	1987
T14	30,661,419/01	2500 kVA, 6.6 kV/11 kV	1987
T15	30,661,421/01	2500 kVA, 6.6 kV/11 kV	1987
T16	30,661,431/01	3.2 MVA, 11 kV, 400 V	2001
T17	30,661,412/01	1250 kVA, 11 kV/400 V	2001
T18	30,661,424/01	1250 kVA, 11 kV/400 V	1999
T19	30,661,425/01	1250 kVA, 11 kV/400 V	2003
T20	30,661,423/01	1250 kVA, 11 kV/400 V	2003
T21	30,661,420/01	2500 kVA, 6.6 kV/11 kV	1998
T22	30,661,416/01	2500 kVA, 6.6 kV/11 kV	1987
T23	30,661,441/01	3.2 MVA, 11 kV, 400 V	1983
T24	30,661,421/01	3.2 MVA, 11 kV, 400 V	2003
T25	30,661,417/01	3.2 MVA, 11 kV, 400 V	1985
T26	30,661,422/01	3.2 MVA, 11 kV, 400 V	1985

A diagnosis procedure was developed to estimate the present status of all transformers. The following tests and analyses were conducted on all transformers.

### Transformer oil diagnostics

- Water content in transformer oil was measured per IEC 60814 Oil-impregnated paper and pressboard^74,75^.
- The breakdown voltage of transformer oil was measured per IEC 60156 Insulating liquids^76,77^.
- The level of acidity present in transformer oil was measured per IEC 62021^78,79^.
- Analysis of corrosive sulfur in transformer oil content using ASTM D1275-15 standard testing procedure for corrosive sulfur in electrical insulating fluids^80^.
- Estimating the water content in insulating paper using the IEC 60814 standard^81,82^.
- DGA of the transformer oil using IEC 60599 standard^83–85^.

### Visual inspection assessments were performed on all accessible transformer components

- Main tank^86^.
- Tap changer^87,88^.
- Oil conservator^89^.
- Breather^90^.
- Cooling tubes^91^.
- Buchholz Relay^92^.

### Analysis of the data consisted of several methods for assessment

Investigation of transformer present state about oil tests and DGA findings:

- Chemical assessments of transformer oil were performed to establish some of its properties that are critical for the mechanical performance of the unit, which covered the following: Chemical properties: (i) water content, (ii) corrosive sulfur, and (iii) acidity number^93^; Electrical properties: (i) dielectric strength, (ii) specific resistance and (iii) dielectric dissipation factor (tan δ)^94,95^. Each of these properties influences the integrity and reliability of transformer oil and its insulating characteristics. In terms of chemical properties, high water concentration in transformer oil may result in lower dielectric strength. The presence of corrosive sulfur in transformer oil creates an acidic environment in the oil, causing accelerated degradation of the cellulose paper and also the oil to become more acidic over time^96^. The quantity of acid present in the transformer is a critical signature that determines transformer oil integrity^97,98^. Electrical properties consist of (i) transformer oil dielectric strength—which signifies the maximum test voltage that the oil will sustain as an insulation material; (ii) specific resistance—which illustrates the insulation characteristics of transformer oil; and (iii) dielectric dissipation factor (tan δ)—indicates the quality of the transformer oil as an insulation material and the level of losses when voltage is applied across it^99–101^.
- Electrical transformers emit decomposition gasses when in service, which are primarily produced from organic insulation. The gas creation process is caused by thermal or electrical challenges, as well as the decomposition of transformer oil or cellulose. This might be due to regular transformer operation or an emergency with the unit in question. A portion of the produced gasses dissolves in the transformer oil^102,103^. DGA is used for evaluating different types of gases in transformer oil and then performing transformer diagnostics. This approach is effective for detecting specific defects (thermal or electrical) and assessing transformer operation^104–106^. There are several techniques for interpreting the DGA. In most cases, a combination of these techniques is utilized to identify the cause of gasses. These approaches include identifying key gases (${\text{H}}_{2}$, ${\text{C}}{\text{H}}_{4}$, ${\text{CO}}$, CO_2_, C_2_H_4_, C_2_H_2_, and ${\text{C}}_{2}{{\text{H}}}_{6}$) as well as their quantities in oil. An increase in the amount of several gases might indicate an issue in the transformer. If this is the case, the gas ratio should be established. Then, to address the issue, an interpretation technique for gas ratios provided in IEC 60599 might be applied. Alternatively, the proposed approach could potentially be utilized for interpreting the data. The values of the obtained gas ratios based on these techniques indicate a specific problem in the performance of the transformer^107^. In summary, the proposed approach has been employed in this work, and it is a good precise approach for transformer diagnosis. It could be utilized as well for periodic testing, with the findings compared over time to monitor the level of gas creation and gauge the actual condition of the transformer^1,3,4,23^.

### Analysis of the condition of the transformers through BPNN algorithms

**Figure 4:**
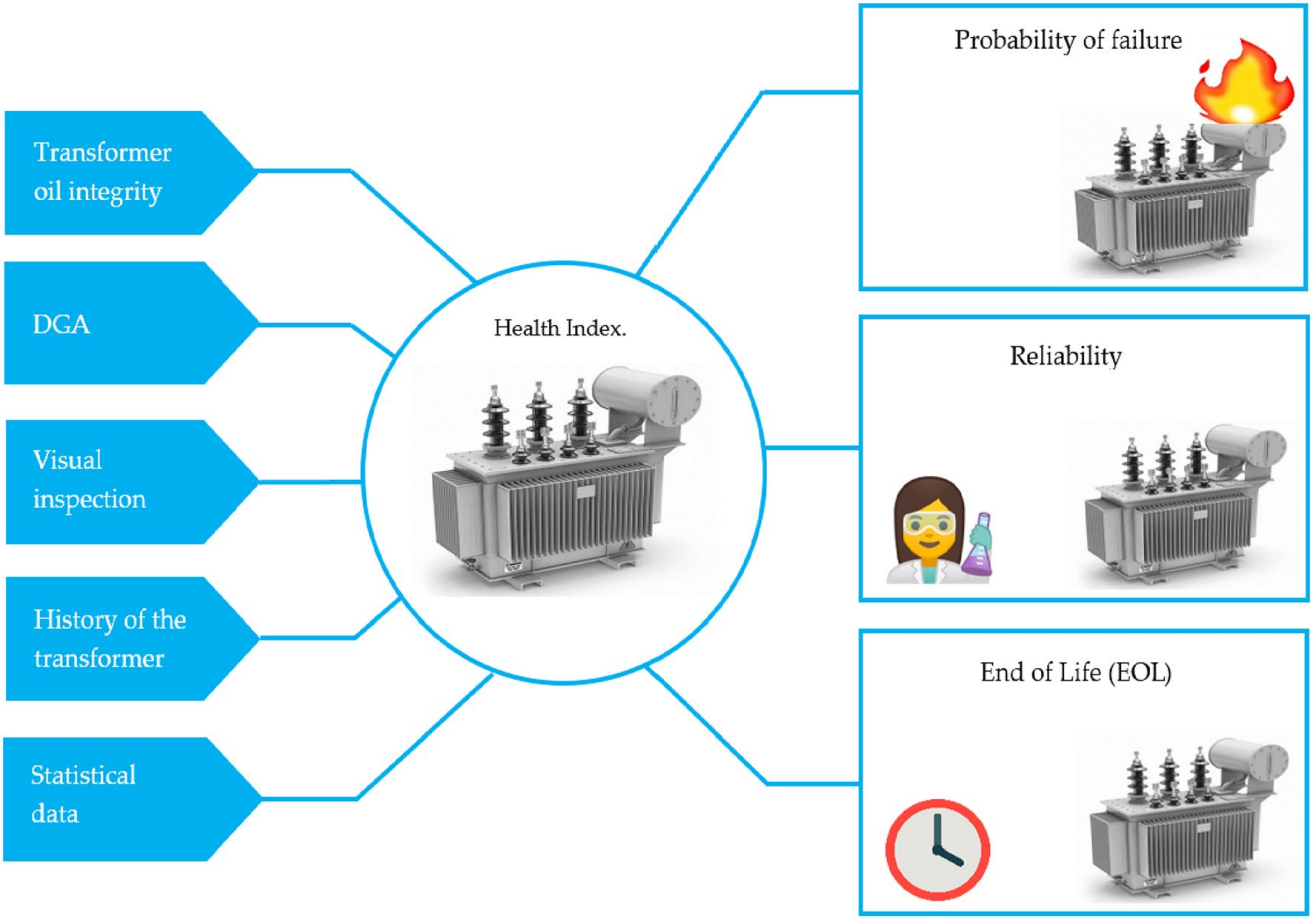
Analysis of LOL.

- This analysis involved two BPNN algorithms for determining the LOL of transformers based on collected and measured data. The first BPNN algorithm proposed is based on forecasting the DP using 2FAL concentration measured from oil samples using DGA, and the second BPNN algorithm proposed is based on forecasting transformer LOL using the 2FAL and DP data obtained from the first BPNN algorithm. To acquire a complete understanding of the state of the transformer, the data based on the BPNN algorithm with weighting coefficients is applied, and the LOL is determined^108–110^. Figure 4 demonstrates the analysis and results of HI components.

Several studies have used the BPNN approach^60,72^ to anticipate numerous transformer states, such as the diagnosis of incipient defects using DGA^111^. In this work, BPNN algorithms are presented to forecast the remaining DP utilizing 2FAL concentration. A databank of 100 samples is utilized to construct the proposed BPNN algorithms using 2FAL as an input and DP as an output. Analysis of the data collected was changed into descriptive data ranging from A “Excellent” to E “Critical” condition^112,113^. DP of cellulose paper^114,115^ was used to estimate the remaining life of the transformer. An accurate estimate of DP to 2FAL was achieved by implementing an algorithm with a BPNN^116,117^. The BPNN algorithms were developed using several transformers and oil samples collected for lab testing. The algorithms were evaluated using cellulose paper samples extracted from transformers that had been removed from service for maintenance and retrofit. Since each transformer has an independent voltage level and capacity, the volume concentrations of dissolved gases in oil vary. Therefore, normalization of the input DGA data was employed to remove the discrepancy. In this work, the relative portion of the oil samples was adopted as the input vectors, which is shown in (1):

1 $${\text{X }}_{{\text{i}}} = \frac{{{\text{X}}_{{{\text{i}}^{*} }} }}{{\sum _{{{\text{j}} = 1}}^{5} {\text{X}}_{{{\text{i}}^{*} }} }}$$

${\text{X}}_{\text{i}}$ denotes the proportion of volume concentration of each gas contained in the oil data. Equation (2) was developed to determine DP using experimental data acquired from transformers well-insulated with standard Kraft paper:

2 $${\text{DP }} = \frac{{{\text{log}}_{{10}} 2{\text{FAL }} - {{ 1}}{{.51}}}}{{ - 0.0035}}$$

Equation (3) was developed to determine DP using experimental data from Kraft cellulose paper as well as hermetical aging tests performed under high-temperature conditions and the degradation of a polymer main chain using (4):

3 $$\text{DP} = \frac{{2.6} - {\text{log}}_{10}2{\text{FAL}}}{0.0049}$$

4 $${\text{DP}} = \frac{{1850}}{{2{\text{FAL}}}} + 2.3$$

Using (5), the DP can be used to forecast the remaining lifespan of the transformer at the time the oil sample was collected. This equation is based on a single variable, specifically the expected DP of cellulose paper.

5 $${\text{Life}}_{{{\text{consumed }}}} = 20{{.5 }} \times {\text{ ln }}\left( {\frac{{1100}}{{{\text{DP}}}}} \right)$$

**Figure 5:**
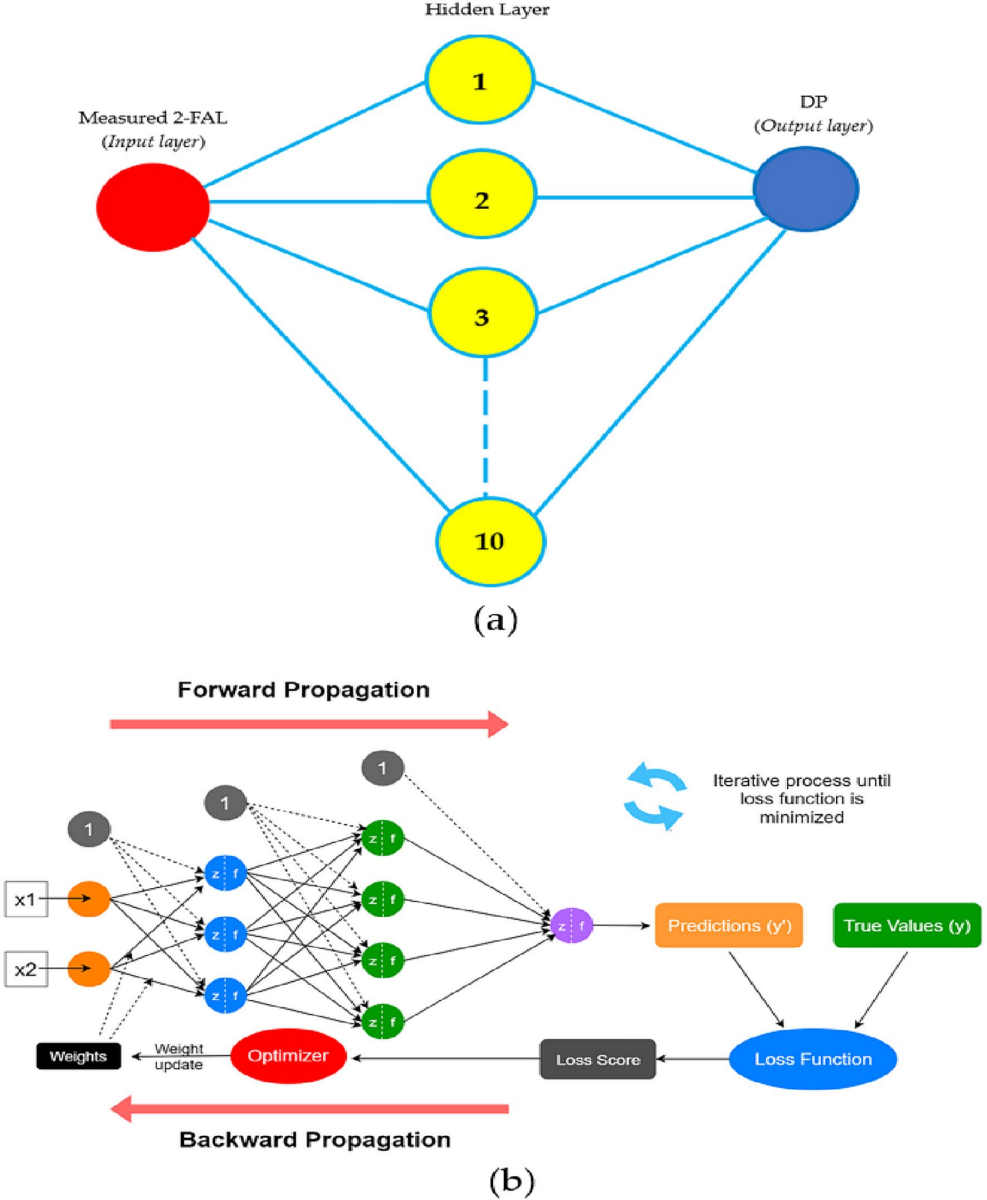
(**a**) Topology of created BPNN for estimation of DP; (**b**) Learning process of a NN.

The BPNN algorithm was adopted in this study due to its capacity to self-learn, simulate non-linear issues, and provide output that is not confined to the input feed, which is important for addressing the issue of cellulose paper for transformer manufacturers^118,119^. Figure 5a illustrates a conceptual representation of the proposed BPNN algorithm for estimating DP. It contains an input layer, hidden layers as required, and an output layer. The system works similarly to a biological neuron, receiving stimuli, interpreting them, and responding with an output. The input layer nodes receive input data and transfer it to the hidden layer 1 nodes via interconnected connections. When data are transported from input nodes to subsequent nodes, they are multiplied with weights and sent to the relevant layer using a transfer function. Similarly, it is forwarded to the output layer, where the target vector is used to compute the error. To generate the precise weighted combination of input data for target vector prediction, weights are altered based on this error. Using an artificial BPNN algorithm has several advantages over more conventional models. It learns the complexities of nature without being explicitly translated into mathematical form. The 2-hidden layers were used for two reasons: (i) the model performs efficiently using the BPNN algorithm, despite the extremely nonlinear input, and (ii) it is unlikely to experience overfitting. The learning process of a neural network (NN) is an iterative process in which the calculations are carried out forward and backward through each layer in the network until the loss function is minimized. This is illustrated in Fig. 5b.

**Figure 6:**
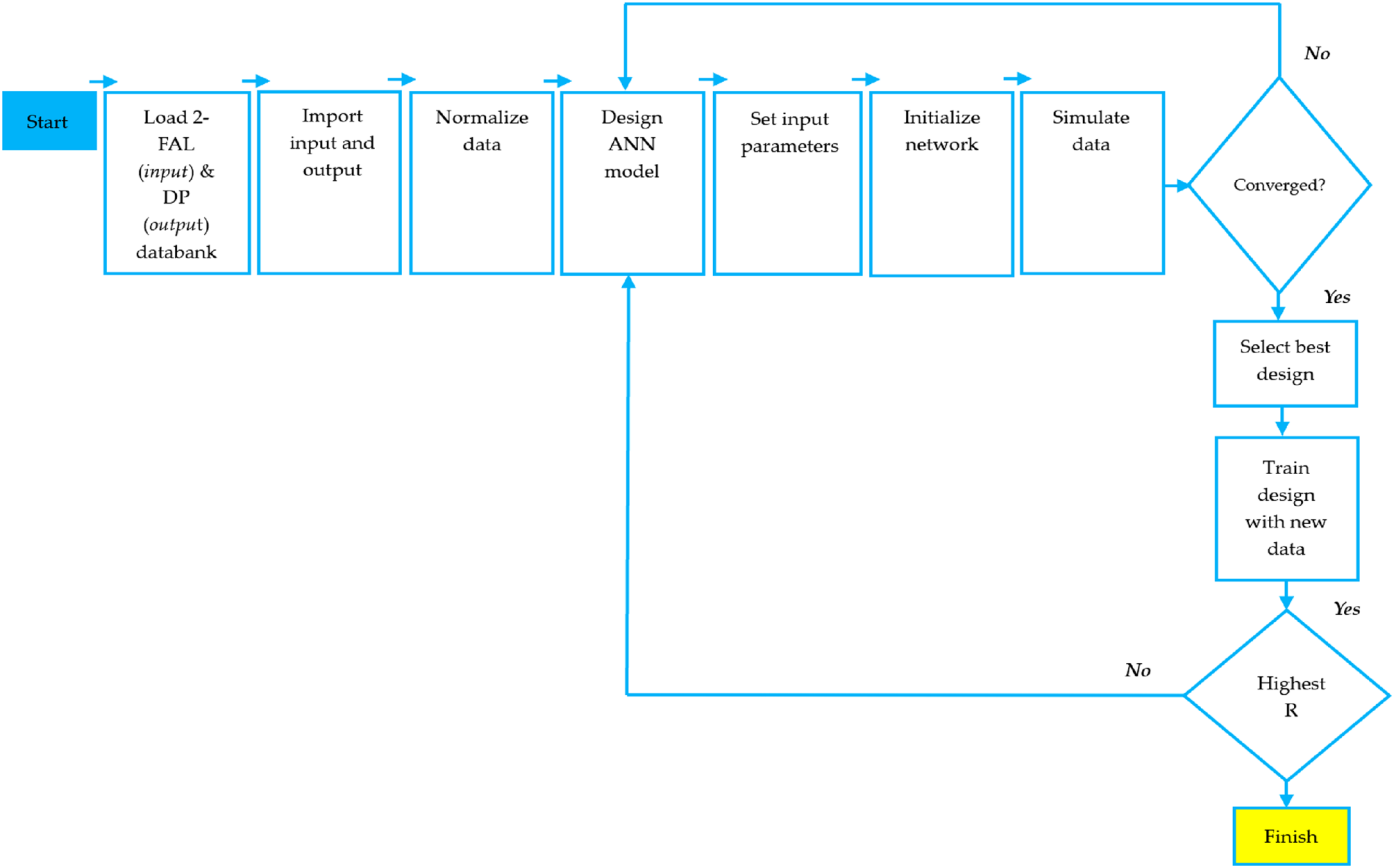
Proposed BPNN flowchart.

The proposed algorithms use the input (2FAL) and the targeted output (DP) supplied to the BPNN network to generate the network output target of a new dataset. Figure 6 shows a detailed flowchart utilized for the design of the proposed BPNN algorithms. The process of designing the BPNN algorithms is divided into three phases: training, testing, and validation^120–122^. The data is prepared using the following steps:

- The preparation of the data ensures that it is error-free, consistent, and has no missing values before it is fed into the BPNN algorithm. These can have an impact on the performance and accuracy of the algorithm, leading to unexpected outcomes.
- The data is modified to match inside the suitable BPNN range. The values are altered to have comparable magnitudes and distributions. This scaling allows the BPNN to learn more quickly and effectively while also avoiding numerical difficulties. To reduce noise creation, normalization, and standardization is used to rescale input and output variables before training the BPNN algorithm^123,124^.
- The data is classified based on labels, classes, or categories and represented numerically using binary encoding for the BPNN to understand. Encoding also aids in reducing the dimensionality and complexity of data. After the data has been cleaned, scaled, and encoded, it is divided into separate sets using *k*-fold cross-validation for training, testing, and validation. This helps to analyze and enhance the BPNN algorithm while avoiding overfitting and underfitting.
- In situations when the BPNN algorithm experiences a shortage of data, data augmentation is employed to improve the size and diversity of the data. Data augmentation involves making numerous transformations and adjustments to existing data, by creating modified copies of a dataset using existing data^125,126^.
- Lastly, data visualization is employed to further study and comprehend the data. Data visualization entails producing graphical interpretations of data, such as charts, graphs, or diagrams. Data visualization aids in identifying patterns, trends, outliers, or correlations in data, as well as providing insights into the BPNN algorithm’s performance.

#### BPNN training

During the BPNN training stage, the network is fed data comprising the 2FAL concentrations and the transformer DP samples as the targeted output. The training stage is essential in the development of the proposed BPNN algorithm. The network dimension, training functionality, adoption learning functionality, number of layers, as well as transfer functionality, are all aspects that have the potential to affect BPNN network efficiency^127^. Moreover, this stage can provide several difficulties, such as overfitting and underfitting^128^. This happens when an algorithm is trained on a large scale of data as it begins to learn from errors and noise in the input data set. Furthermore, testing using test data that has a high variance. The algorithm then fails to appropriately classify the data due to too many details and noise, and it leads to bad performance of the network. Stopping the network early has been utilized to prevent network overfitting and underfitting^129^. The optimal BPNN settings with the maximum precision, which is equivalent to the correlation coefficient (*R*), were obtained by modifying the number of hidden layers, the number of neurons, as well as the transfer functionality^108,130^. In this work, a two-layer system with 10-hidden layers and a 1-output layer was adopted for both BPNN algorithms. The proposed BPNN algorithm was developed using a databank containing 100 data sets, of which 70 were utilized for training and 30 for testing and validation. While a 1-hidden layer is sufficient for nonlinear modeling, a system with 2-hidden layers outperforms systems with 1-hidden layer in terms of the number of iterations, precision, and complexity. Furthermore, the 2-layer system helps solve the challenge of slow learning rates^73^.

#### BPNN testing

**Figure 7:**
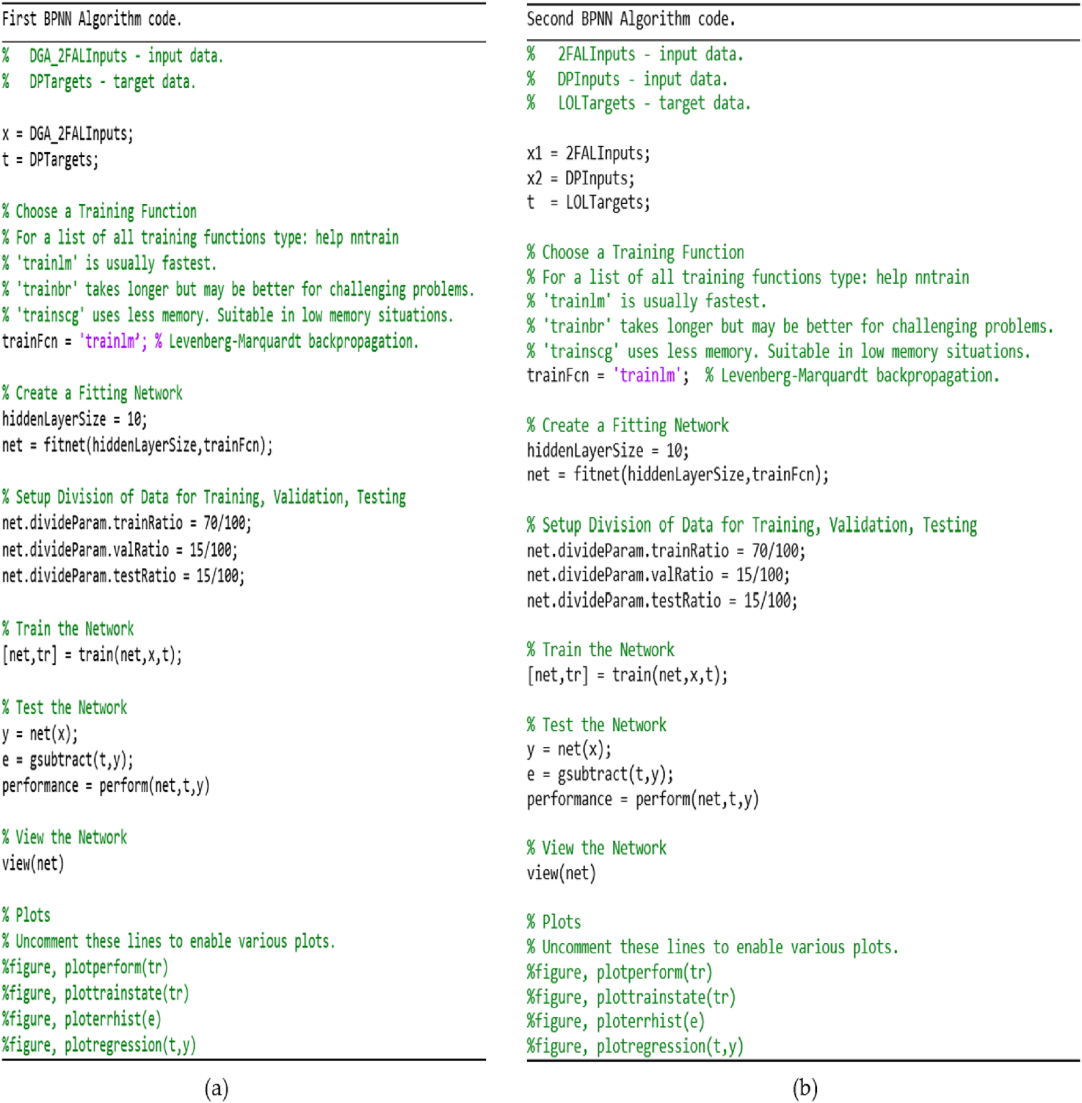
(**a**) BPNN coding to obtain DP using 2FAL; (**b**) BPNN coding to forecast LOL using DP and 2FAL.

**Figure 8:**
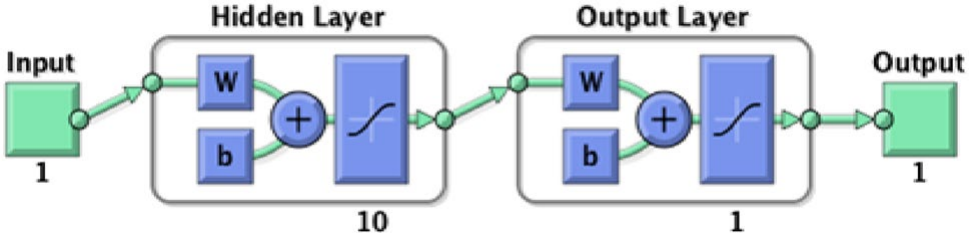
The BPNN configuration for DP estimation.

**Figure 9:**
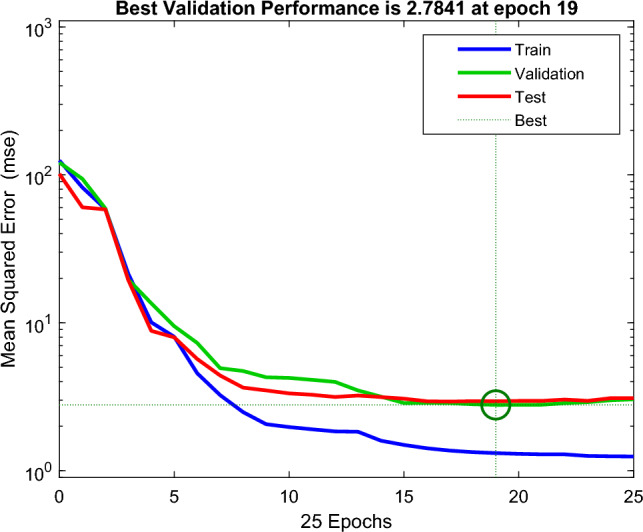
Mean Square Error (MSE) plot of the first BPNN algorithm.

During BPNN testing, an unknown dataset is added to the network to evaluate the efficiency of the trained network. At this stage, the BPNN network is evaluated using linear regression modeling. The *R* is determined to analyze the relationship between the BPNN network outputs and the intended output. A successful network will have an *R*-value close to 1, indicating that there is a notable relationship between the BPNN network output and the targeted output. The overall efficiency of the BPNN network is expressed by the value of *R* and the best BPNN network is identified based on its closest relationship value to 1^68,95^. The Levenberg–Marquardt (LM) training approach^64,65^ was used since it is recommended as the preferable supervised algorithm in the MATLAB environment due to its fast training rate^116^. Figure 7a,b show the coding of the two algorithms. Figure 8 shows the modeling configuration that predicts DP using only 2FAL concentration. Figure 9 shows the Mean Square Error (MSE)^94^ plot of the first BPNN algorithm. The three curves represent the change in the MSE with epochs for training, validation, and testing. However, the best algorithm was reached at epoch 19 as marked by the vertical dash line. It proves that the network achieved better results during the training stage compared to the testing stage because the desired outputs of the test data are always unknown to the network. No significant overfitting occurred with this iteration. The characteristics of both the validation and test curves are similar. The remarks above indicate acceptable results for the network.

**Figure 10:**
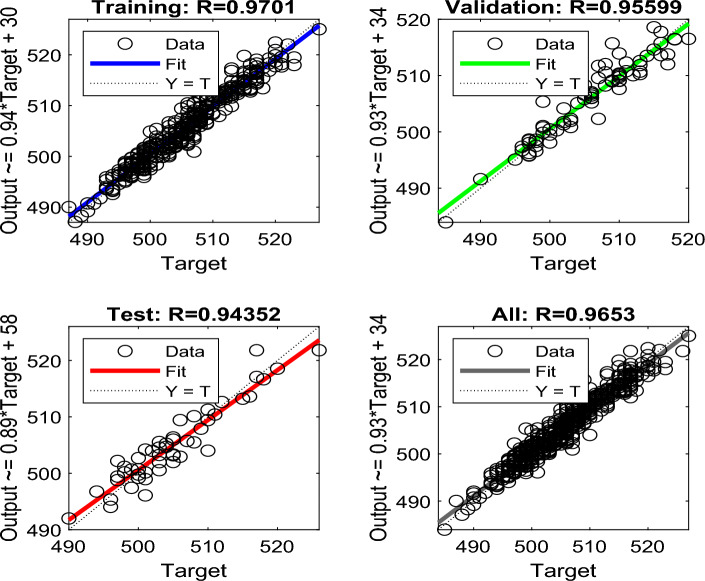
BPNN performance results for the estimation of DP from 2FAL.

Figure 10 shows the regression analysis of the NN modeling of the training, validation, test, and complete datasets presented in this study. The training dataset consists of 100 data points (i.e. 70% of total data points for training), while the validation and test datasets have 30 data points (i.e. 15% each). It can be observed that the algorithm produces a correlation coefficient of 0.970 for the training, 0.956 for the validation, 0.944 for the testing, and an overall correlation of 0.965 when the DP is predicted using the 2FAL measured in oil. From Fig. 10, it is observed that the four linear fit lines were achieved (Eqs. 6–9) in the form $\text{y = a.x + b}$, where *x* and *y* are observed and predicted peak ground acceleration (PGA), respectively, which are listed below:

6 $$\text{y} = 0.970.x + 30$$

7 $$\text{y} = 0.956.x + 58$$

8 $$\text{y} = 0.944.x + 34$$

9 $$\text{y} = 0.965.x + 34$$

**Figure 11:**
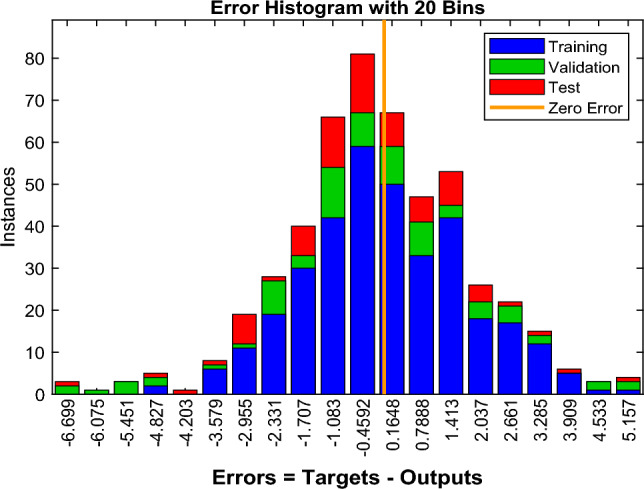
Error histogram of the first BPNN algorithm.

Figure 11 shows the error histogram for our NN modeling showing most of the larger positive and negative values are near the zero-error line (*shown by an orange thick line*), which suggests the error distribution of the NN modeling is good. Thus, the algorithm is well trained.

**Figure 12:**
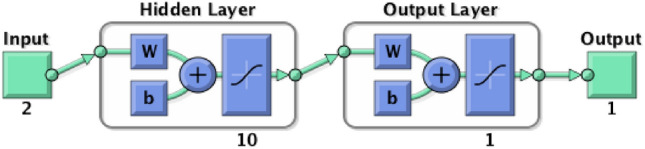
The BPNN configuration for LOL forecasting.

**Figure 13:**
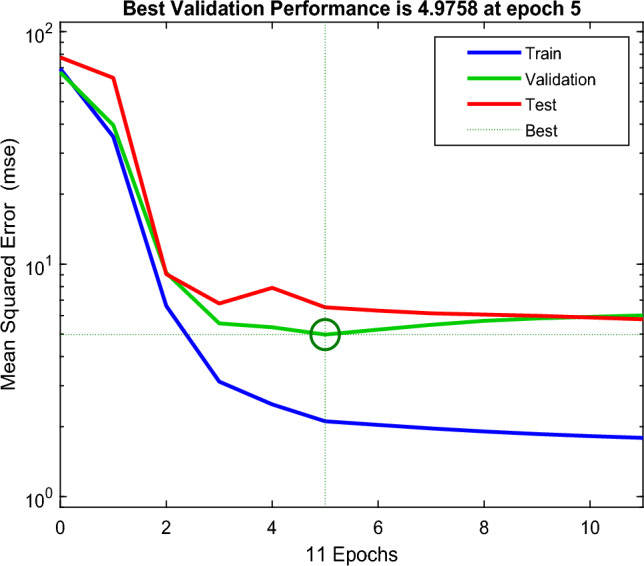
Mean Square Error (MSE) plot of the second BPNN algorithm.

Figure 12 illustrates the algorithm setup for forecasting LOL based on predicted DP and measured 2FAL. Figure 13 shows the MSE plot of the first BPNN algorithm. The network produces a substantial MSE at first, however, it decreases as the training advances to 5 epochs. As demonstrated in Fig. 13, the best validation performance of the second BPNN algorithm occurred at 5 iterations, as shown by the vertical dash line. This iteration shows a minor overfitting. The characteristics of both the validation and test curves are slightly different; however, the remarks above indicate acceptable results for the network.

**Figure 14:**
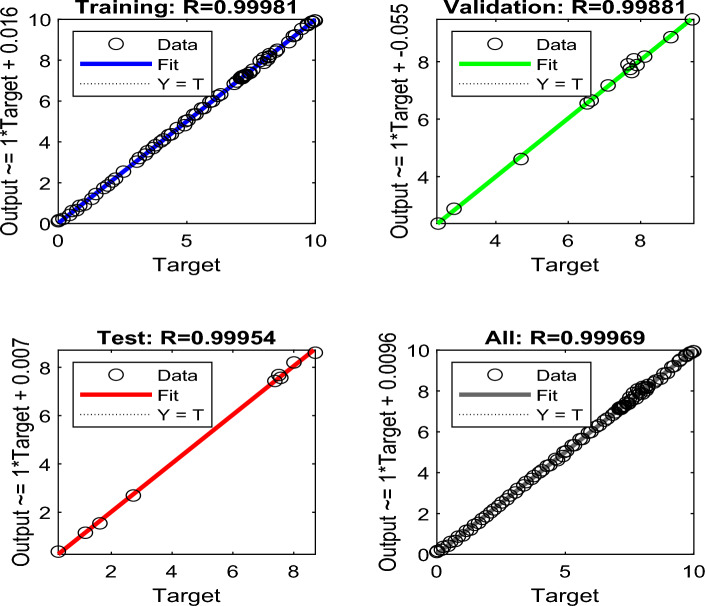
BPNN performance results for forecasting LOL from 2FAL and DP.

Figure 14 shows the second proposed BPNN algorithm performance plot, and the regression analysis of the NN modeling for our training, validation, test, and complete datasets. The training dataset consists of 100 data points (i.e. 70% of total data points), while the validation and test datasets have 30 data points (i.e. 15% each). It can be observed that the algorithm produces a correlation coefficient of 0.999 for the training, 0.999 for the validation, 0.999 for the testing, and an overall correlation of 0.999 when the prediction of transformer LOL concerning 2FAL generated and the amount of DP present produced. From Fig. 14, it is observed that the four linear fit lines were achieved (Eqs. 10–13) in the form $y=a.x+b$, where *x* and *y* are observed and predicted PGA, respectively, which are mentioned below:

10 $$\text{y} = 0.999.x + 0.016$$

11 $$\text{y} = 0.999.x + 0.007$$

12 $$\text{y} = 0.999.x + 0.055$$

13 $$\text{y} = 0.999.x + 0.096$$

**Figure 15:**
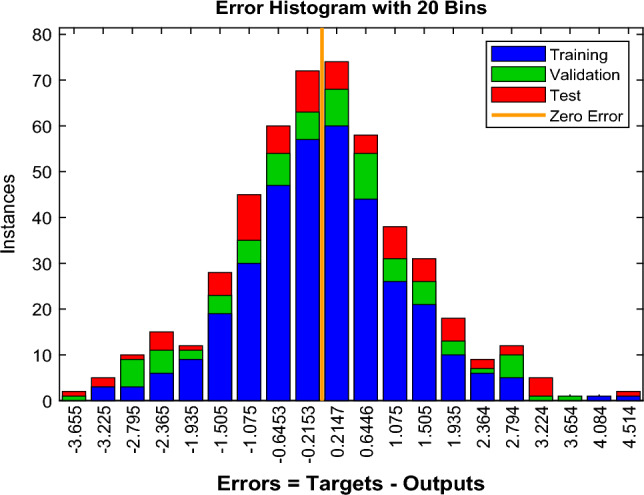
Error histogram of the second BPNN algorithm.

**Figure 16:**
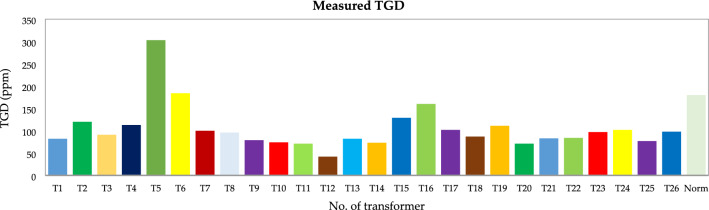
Measured total gas dissolved (TGD).

Figure 15 shows the error histogram for our NN modeling showing most of the larger positive and negative values are near the zero error line (shown by an orange thick line), which suggests the error distribution of the NN modeling is good. Thus, the algorithm is well trained. Figure 16 is given as some results of trained neurons, and the overall amount of gas dissolved in the oil, which is the average of all gas amounts recorded.

Table 6 summarizes the analytical settings used in the proposed BPNN algorithms. The MSE, *R*, and computation time for the two algorithms are contrasted in Table 7. The second algorithm outperforms the first algorithm in terms of *R*. Due to the large quantity of data to train, the second approach takes a bit more time to compute than the first algorithm. However, because the simulation is done offline, the computation time cannot be used to determine the optimum algorithm^44^. Consequently, after considering all simulation data, it is proven that the second algorithm has an exceptional capacity to accurately forecast transformer LOL^131^.

**Table 6:** BPNN algorithm settings.

Sample	2FAL
Data division	Random
Training	Levenberg–Marquardt
Performance	Mean Square Error
Calculations	MEX

**Table 7:** Comparison of the two proposed BPNN algorithms.

Index	MSE	*R*	Computation time	Rank
BPNN 1	2.7841 × 10^3^	0.965	23.4	2
BPNN 2	4.9758 × 10^2^	0.999	26.2	1

Transformer lifespan estimates were performed using the calculated HI coefficient. The HI coefficients and grades are presented in Table 8. The integrity of the transformer was specified as its capacity to withstand minimal loads and pressures while preserving its functional specifications over time. Transformer condition reliability can be calculated using (14):

14 $$R_{i} (t) = e^{ - \lambda t}$$

where* t* is the time in years, and $\lambda = f(HI)$

**Table 8:** HI coefficients and grades.

Index	Specifications	*t*	Grades	λ
1	DGA	15	A, B, C, D	4, 3, 2, 1……
2	Load history	6	A, B, C, D	4, 3, 2, 1……
3	Temperature and hotspots	4	A, B, C, D	4, 3, 2, 1……
3	Oil integrity	15	A, B, C, D	4, 3, 2, 1……
5	Furans	9	A, B, C, D	4, 3, 2, 1……
6	Cellulose paper	5	A, B, C, D	4, 3, 2, 1……
7	Water content in cellulose paper	5	A, B, C, D	4, 3, 2, 1……
8	Main and conservator tank condition	4	A, B, C, D	4, 3, 2, 1……
9	Tap changer	5	A, B, C, D	4, 3, 2, 1……
10	Cooling system and Explosion vent	3	A, B, C, D	4, 3, 2, 1……
11	Buchholz relay condition	6	A, B, C, D	4, 3, 2, 1……
12	Rust	4	A, B, C, D	4, 3, 2, 1……
13	Oil leakage and level	2	A, B, C, D	4, 3, 2, 1……
14	Grounding system	2	A, B, C, D	4, 3, 2, 1……
15	Paint	8	A, B, C, D	4, 3, 2, 1……
16	Working clearance	7	A, B, C, D	4, 3, 2, 1……

When a transformer is operating normally, without external or sudden issues that might cause failure, all parts of the equipment deteriorate at a constant rate, which is usually specified by its working years and the current condition of all the parts. To account for all of the transformer’s parts that have an impact on its condition over extended periods, and years, the HI is included in the calculation of the transformer’s durability. The formula used to determine the failure rate is given in (15):

15 $$\lambda = \frac{1 - HI}{{A^{2} }}$$

where parameter $A = f (t, \gamma )$. Parameter A is calculated by using (16):

16 $$A = \frac{{t_{e} - t}}{y}$$

where *t* is the years of service of the transformer; γ is coefficient 1, 2, 3, and 4 depending on the degrading curve (γ = 1 if the transformer is relatively new and it is in the zone of sudden failures; γ = 2 if the transformer is working normally but its designed working years are unknown; γ = 3 when the transformer has already exceeded its initially planned years of service; γ = 4 when the transformer is a risk); and ${\text{t}}_{\text{e}}$ is the expected working years of the transformer. All calculations were performed with ${\text{t}}_{\text{e}}$ = 61 years. Durability was used to calculate the risk of failure. Transformer durability and risk of failure statistics are shown in the estimated years that the transformers will operate reliably.

## Results and analysis

The outcomes of all conducted experiments and proposed BPNN algorithms are reported for all evaluated transformers, ranging from T1 to T26. The data were collected using transformer oil sampling and DGA. Sampling was performed to predict the water concentration in cellulose paper based on chemical analysis for oil integrity. Data was obtained using visual inspections as well as the past events of the transformer, which was investigated during the testing process. The outcomes of the proposed BPNN algorithms were established on HI estimations, predicted transformer lifespan based on estimated DP via the BPNN, and durability and risk of failure estimates based on the HI. The results gathered from all conducted checks and analyses on all studied transformers were consolidated.

### Transformer oil measurements and test data

#### DGA of the transformer oil

Table 9 illustrates a description of the investigated gases discussed in the study and Table 10 shows the measurements of dissolved gases detected and recorded in oils of the evaluated transformers.

**Table 9:** Description of gases.

Gas detected	Description
${\text{C}}_{2}{{\text{H}}}_{2}$	Acetylene
${\text{C}}_{2}{{\text{H}}}_{6}$	Ethane
${\text{C}}_{2}{{\text{H}}}_{4}$	Ethylene
${\text{CH}}_{4}$	Methane
${\text{CO}}$	Carbon monoxide
${\text{CO}}_{2}$	Carbon dioxide
${\text{H}}_{2}$	Hydrogen
${\text{O}}_{2}$	Oxygen

**Table 10:** Measured gases in ppm from DGA for investigated transformers.

Index	${\text{H}}_{2}$	${\text{CH}}_{4}$	${\text{C}}_{2}{{\text{H}}}_{2}$	${\text{C}}_{2}{{\text{H}}}_{4}$	${\text{C}}_{2}{{\text{H}}}_{6}$	${\text{CO}}$	${\text{CO}}_{2}$	${\text{O}}_{2}$
T1	6	3	0.5	10	9	53	722	NA
T2	6	3	0.5	2	18	86	756	NA
T3	6	3	7.5	17	3	70	687	NA
T4	8	3	9	21	3	83	703	NA
T5	8	3	116.4	3	5	103	945	NA
T6	6	3	57.8	6	5	75	798	NA
T7	6	15	4	5	3	68	766	NA
T8	6	7	3	5	3	55	821	NA
T9	8	5	0.6	5	3	66	939	NA
T10	9	3	0.5	16	3	67	963	NA
T11	8	13	0.5	11	2	49	872	NA
T12	8	3	0.5	6	3	33	537	NA
T13	7	3	0.5	15	2	39	671	NA
T14	6	3	11.3	64	4	41	825	NA
T15	6	2	9	31	3	40	899	NA
T16	6	2	12.5	17	4	91	891	NA
T17	6	5	21.6	4	6	73	567	NA
T18	8	2	11	18	5	29	731	NA
T19	8	3	0.7	13	4	31	723	NA
T20	6	3	0.6	3	3	52	901	NA
T21	6	1	0.6	12	3	31	875	NA
T22	6	1	0.8	41	3	57	633	NA
T23	6	3	0.5	33	2	65	800	NA
T24	7	3	0.5	2	5	111	804	NA
T25	7	4	2	5	6	89	540	NA
T26	8	11	6.3	19	5	37	981	NA

#### Chemical analysis of transformer oil

**Figure 17:**
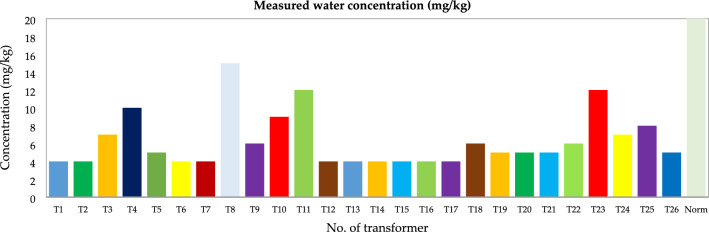
Water concentration (mg/kg).

**Figure 18:**
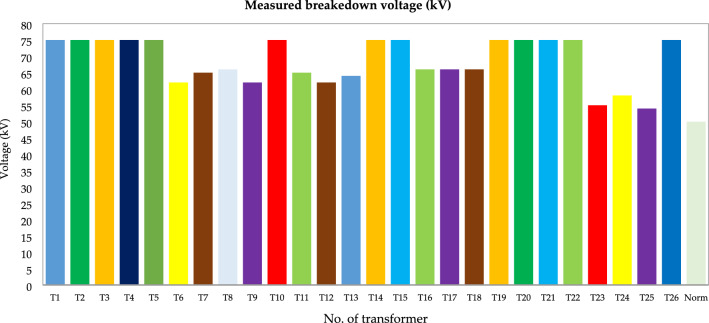
Breakdown voltage (kV).

**Figure 19:**
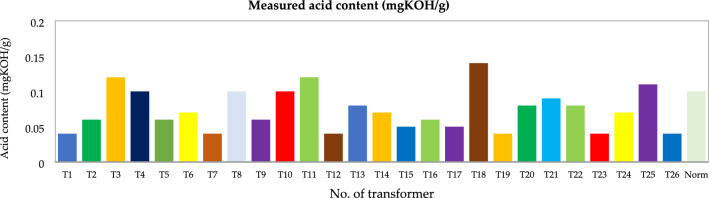
Acid content (mgKOH/g).

**Figure 20:**
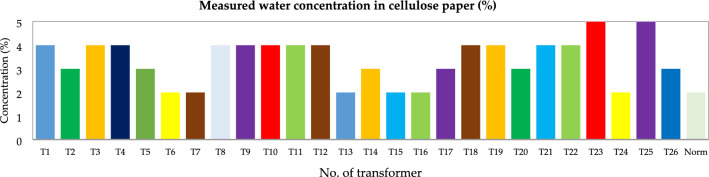
Water concentration in cellulose paper (%).

Figures 17, 18, 19 and 20 illustrate the chemical and electrical properties of the evaluated oil data.

### Data collected through visual inspection

#### Main tank

Table 11 provides an overview of the information gathered from visual inspections of the main tanks of all the transformers under investigation.

**Table 11:** Defects identified during visual inspections of main tanks.

Inspected defect	No. of transformers with defects	Transformers without noted defects
Exterior of unit	9	T2, T3, T4, T5, T6, T9, T10, T11, T12, T14, T15, T18, T19, T22, T23, T24, T25
Proper grounding	15	T3, T4, T5, T6, T16, T17, T19, T20, T22, T25, T26
Rust	26	–
Seals	5	T1, T2, T3, T4, T5, T6, T9, T10, T11, T12, T13, T15, T16, T17, T18, T19, T20, T21, T22, T25, T26
Oil level, leakage, thermometer and drain valve	17	T8, T9, T10, T16, T17, T19, T20, T21, T23
Paint	21	T9, T10, T11, T12, T13, T26
Working clearance	0	T1, T2, T3, T4, T5, T6, T7, T8, T9, T10, T11, T12, T13, T14, T15, T16, T17, T18, T19, T20, T21, T22, T23, T24, T25, T26

#### Tap changer

Table 12 provides an overview of the information gathered from visual inspections of the tap changers of all the transformers under investigation.

**Table 12:** Defects identified during visual inspections of tap changers.

Inspected defect	No. of transformers with defects	Transformers without noted defects
Oil leakage	1	T1, T2, T3, T5, T6, T7, T8, T9, T11, T12, T13, T14, T15, T16, T17, T18, T19, T20, T21, T22, T23, T24, T26
Loose connections	4	T2, T3, T4, T5, T6, T7, T8, T9, T11, T12, T13, T14, T16, T17, T18, T19, T20, T22, T23, T24, T25, T26
Covers	5	T1, T2, T3, T4, T5, T6, T9, T10, T11, T12, T13, T15, T16, T17, T18, T19, T20, T21, T22, T25, T26
Proper operation	4	T2, T3, T4, T5, T6, T7, T8, T9, T11, T12, T13, T14, T16, T17, T18, T19, T20, T22, T23, T24, T25, T26
Working clearance	0	T1, T2, T3, T4, T5, T6, T7, T8, T9, T10, T11, T12, T13, T14, T15, T16, T17, T18, T19, T20, T21, T22, T23, T24, T25, T26

#### Oil conservator

Table 13 provides an overview of the information gathered from visual inspections of the oil conservators of all the transformers under investigation.

**Table 13:** Defects identified during visual inspections of oil conservator tanks.

Inspected defect	No. of transformers with defects	Transformers without noted defects
Rust	11	T1, T8, T9, T10, T11, T12, T13, T14, T17, T18, T19, T20, T21, T22, T23
Seals	5	T1, T2, T3, T4, T5, T6, T9, T10, T11, T12, T13, T15, T16, T17, T18, T19, T20, T21, T22, T25, T26
Oil leakage	17	T8, T9, T10, T16, T17, T19, T20, T21, T23
Paint	21	T9, T10, T11, T12, T13
Working clearance	0	T1, T2, T3, T4, T5, T6, T7, T8, T9, T10, T11, T12, T13, T14, T15, T16, T17, T18, T19, T20, T21, T22, T23, T24, T25, T26

#### Breather

Table 14 provides an overview of the information gathered from visual inspections of the breathers of all the transformers under investigation.

**Table 14:** Defects identified during visual inspections of breathers.

Inspected defect	No. of transformers with defects	Transformers without noted defects
Silica gel color	25	T22
Breathing holes	1	T1, T2, T3, T4, T5, T6, T9, T10, T11, T12, T13, T15, T17, T18, T19, T20, T21, T22, T25, T26
Cap oil level	24	T22, T26
Cracks	3	T1, T2, T3, T4, T6, T7, T8, T9, T10, T12, T13, T14, T15, T16, T17, T18, T19, T20, T21, T22, T24, T25, T26
Working clearance	0	T1, T2, T3, T4, T5, T6, T7, T8, T9, T10, T11, T12, T13, T14, T15, T16, T17, T18, T19, T20, T21, T22, T23, T24, T25, T26

#### Cooling tubes

Table 15 provides an overview of the information gathered from visual inspections of the cooling tubes of all the transformers under investigation.

**Table 15:** Defects were identified during visual inspections of cooling tubes.

Inspected defect	No. of transformers with defects	Transformers without noted defects
Rust on cooling tubes	26	–
Rust on cooling fans	26	–
Oil leakage on tubes & valves	26	–
Missing tubes & coolers	0	T1, T2, T3, T4, T5, T6, T7, T8, T9, T10, T11, T12, T13, T14, T15, T16, T17, T18, T19, T20, T21, T22, T23, T24, T25, T26
Missing fans	0	T1, T2, T3, T4, T5, T6, T7, T8, T9, T10, T11, T12, T13, T14, T15, T16, T17, T18, T19, T20, T21, T22, T23, T24, T25, T26

#### Buchholz relay

Table 16 provides an overview of the information gathered from visual inspections of the Buchholz relays of all the transformers under investigation.

**Table 16:** Defects identified during visual inspections of Buchholz relays.

Inspected defect	No. of transformers with defects	Transformers without noted defects
Missing magnetic oil level indicator	0	T1, T2, T3, T4, T5, T6, T7, T8, T9, T10, T11, T12, T13, T14, T15, T16, T17, T18, T19, T20, T21, T22, T23, T24, T25, T26
Faulty magnetic oil level indicator	3	T1, T2, T3, T4, T5, T6, T7, T8, T9, T10, T11, T12, T13, T14, T15, T16, T17, T18, T19, T20, T21, T22, T26
Missing oil temperature indicator	1	T1, T2, T3, T4, T5, T6, T8, T9, T10, T11, T12, T13, T14, T15, T16, T17, T18, T19, T20, T21, T22, T23, T24, T25, T26
Faulty oil temperature indicator	0	T1, T2, T3, T4, T5, T6, T7, T8, T9, T10, T11, T12, T13, T14, T15, T16, T17, T18, T19, T20, T21, T22, T23, T24, T25, T26

### Results obtained through applied analysis of collected data

#### DGA of the transformer oil and oil analysis

Table 17 provides an overview of the conclusions drawn from the analysis of the data.

**Table 17:** Summary of the DGA and oil test analysis.

Index	DGA results	Oil test results
T1	No fault detected	Normal
T2	No fault detected	Normal
T3	Fault was detected with low partial discharges and overheating with temperature below T < 300 °C	Acid content high
T4	No fault was detected. High quantities of methane ${\text{CH}}_{4}$, caution is needed	Acid content high
T5	No fault detected	Normal
T6	No fault detected	Normal
T7	No fault detected	Normal
T8	No fault was detected. High quantities of methane ${\text{CH}}_{4}$, caution is needed	Acid content high
T9	No fault detected	Normal
T10	Fault was detected with low partial discharges and overheating with temperature below T < 300 °C	Acid content high
T11	Fault was detected with low partial discharges and overheating with temperature below T < 300 °C	Acid content high
T12	No fault detected	Normal
T13	No fault detected	Normal
T14	No fault detected	Normal
T15	No fault detected	Normal
T16	No fault detected	Normal
T17	No fault detected	Normal
T18	No fault detected	Normal
T19	No fault detected	Normal
T20	No fault detected	Normal
T21	No fault detected	Normal
T22	No fault detected	Normal
T23	No fault was detected. High quantities of methane ${\text{CH}}_{4}$, caution is needed	Acid content high
T24	No fault detected	Normal
T25	Fault was detected with low partial discharges and overheating with temperature below T < 300 °C	Acid content high
T26	No fault detected	Normal

#### Health Index (HI)

**Figure 21:**
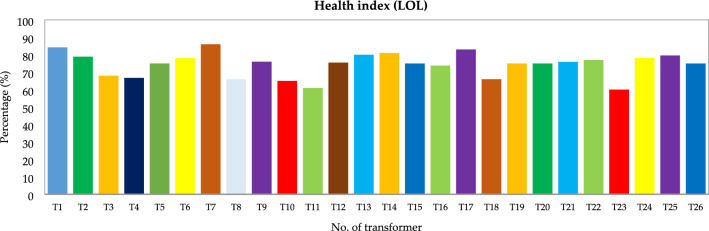
Calculated HI for transformers T1–T26.

Figure 21 illustrates the outcomes of the HI calculations that were performed using the algorithms proposed in in study.

Table 18 shows the estimated years of operation for each transformer according to the calculated HI. It was estimated that six transformers would last more than 15 years. There were no transformers discovered to have near EOL or EOL materials. Small to medium-sized defects that can be resolved to stop serious failures were discovered in every transformer. The service and maintenance of seven transformers were given priority.

**Table 18:** HI values and expected years of service.

Health index (%)	Condition	Expected years of service	No. of transformers
85–100	Excellent	> 15	5
70–85	Good	> 10	14
50–70	Fair	< 10	7
30–50	Poor	< 3	0
0–30	Critical	End of Life	0

#### Calculated degree of polymerization

**Figure 22:**
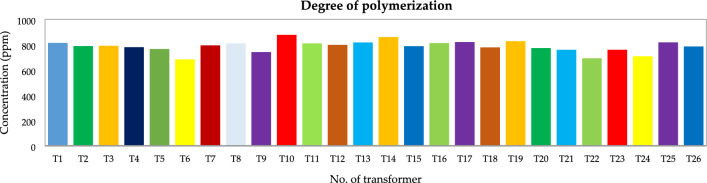
Estimated DP for transformers T1–T26.

Table 19 displays the compiled results for all transformers, and Figure 22 displays the estimated DP for all units using the proposed BPNN algorithms. The results demonstrate that none of the units was discovered to be approaching the end-of-life category, and all tested units meet the standard aging domain of cellulose paper^132^.

**Table 19:** Evaluation of DP and cellulose paper properties.

Concentration (ppm)	Condition	No. of transformers
600–800	Normal aging rate	26
360–600	Accelerated aging rate	0
300–360	Excessive aging rate	0
200–300	High risk of failure	0
< 200	End of life	0

To improve estimation precision and diagnose defect types, diagnostic techniques must be classified based on their susceptibility and monitoring capabilities against defects and insulation deterioration. Table 20 summarizes routine and diagnostic analysis according to their capacity to discover defects. According to IEEE C57.104^133^, DGA alone is capable of diagnosing 70% of typical defects, however, additional evaluations are required to identify mechanical defects.

**Table 20:** Comparison between online, routine, and diagnostic analysis for transformer condition.

Diagnose name	Online monitoring	Routine analysis	Diagnostic analysis	Defects detection types		
				Electrical	Mechanical	Thermal
DGA	X	X	X	X		X
Oil sampling		X	X	X		X
Furan sampling		X	X	X		X
DP assessment			X	X		
Partial discharge (PD) diagnostic	X		X	X		
Cellulose analysis		X	X	X	X	X
Chemical analysis	X	X	X	X		X
Leakage reactance			X		X	
Insulation resistance		X		X		
Winding resistance		X				X
Sweep frequency response analysis (SFRA)		X	X		X	
HI			X	X		X
Visual inspection		X			X	

To improve dependability, significant industry regulations and improved research, such as CIGRE, IEEE, and IEC, have been applied to specific procedures for evaluating and analyzing test results. These quantitative and diagnostic procedures can assist maintenance professionals in interpreting test results and recommending key transformer characteristics that ought to be monitored. These procedures can assist utilities in preventing unforeseen breakdowns and offer a rationale for plant managers for the replacement of unreliable aging transformers through accurate forecasts. This work offers an overview of current testing as well as transformer condition assessment strategies. This is a discipline whereby a great deal of research is being conducted to better comprehend the features of various tests and to develop improved techniques for integrating test results to monitor the state of this costly and vital equipment. As the number of units of assets ages, the relevance of these strategies appears to increase even more.

## Discussion

Electrical transformers are essential to the electrical system, and detecting latent defects immediately can help prevent more significant issues. This work tackled the issue of predicting and identifying electrical transformer defects using DGA data samples that result from low-occurring transformer issues. The capability to monitor equipment degradation opens up possibilities of reducing costs related to repairs and maintenance while also avoiding a variety of unexpected events. This will enable long-term studies on invested materials as well as a reduction in the initial energy required to design new units. The twenty-six electrical transformers, which were the topic of complex evaluations and inspection, were addressed and classified into different classes based on their HI, DP, DGA, and *P* (*risk of failure*). These classes involved scheduled maintenance and repair. The HI observations showed that the units had a significant amount of residual life and the technical capability of being in operation for extra years. As a consequence, none of the transformers required to undergo repair. The findings demonstrate that the transformers T1, T2, T5, T6, T7, T8, T9, T12, T13, T14, T15, T16, T17, T18, T19, T20, T21, T22, T24, and T26 are operating normally, thus no preventative action required at this stage. Methane levels in T4, T8, and T25 were approaching alert limits and were planned to undergo further diagnostics and monitoring. DGA discoveries in transformers T3, T10, T11, and T25 led to the diagnosis of an electrical fault and were planned to undergo visual inspection and testing. Transformers T3, T5, and T17 produced positive HI and DP findings, however, the DGA and *R* results indicate that cautious/or further diagnostics are required and the units were scheduled for moisture purification and resampling for safety and economic purposes. Table 21 summarizes the findings of the complex inspections and evaluations of the twenty-six electrical transformers surveyed, with color-coded data.

**Table 21:**
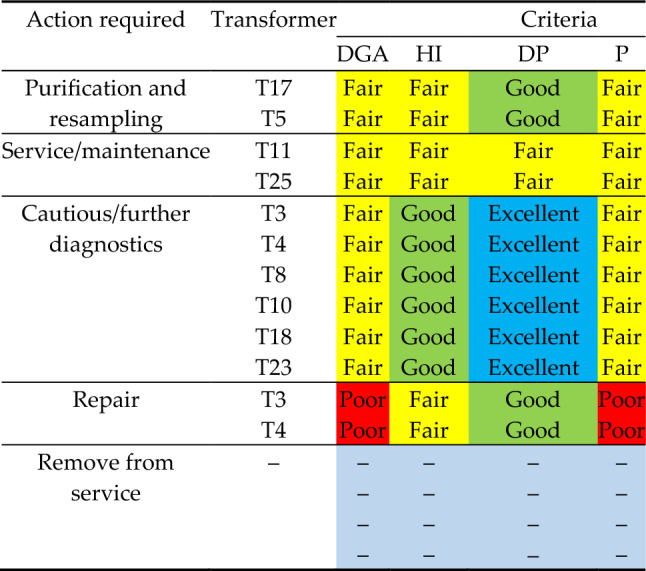
Required action for the investigated transformers.

In this study, the approaches together supplied more detailed knowledge on the condition of each transformer as well as its expected lifespan. As per the 2FAL results obtained from the oil data, each transformer had minimal insulation degradation as well as a significant DP. However, their expected lifespans over the years of operation differed, and according to the HI, eighteen units performed admirably. Table 22 illustrates comparisons derived from all diagnostic techniques applied. The actions that must be implemented when utilizing DP data to assess the condition of the transformer to implement safety precautions are listed in Table 23.

**Table 22:** Grade comparisons for the four assessments conducted.

Techniques employed	No. of transformers			
	Excellent	Good	Fair	Poor
DGA	18	6	2	0
BPNN algorithms (2FAL, DP)	18	6	2	0
Health index	5	14	7	0
Visual inspection	0	16	8	2

**Table 23:** Recommendations for transformer measures based on results.

DP value	Comment
< 200	The test results show substantial cellulose paper deterioration that exceeds the critical threshold. It is highly recommended that the unit be removed from service immediately and visually inspected
200–250	The cellulose paper is close to or in a catastrophic state. Recommended that the unit be removed from service and properly examined as soon as feasible. Direct DP analysis can be performed on paper samples
260–350	The cellulose paper is nearing the end of its life. To review the state, schedule a verification and/or re-sample within 1 year
360–450	The cellulose paper is approaching its critical state. Recommend re-sampling in 1–2 years
460–600	Substantial cellulose paper degradation, however, is still far from the critical threshold
610–900	Low to moderate cellulose paper aging
> 900	No observable deterioration of the cellulose paper

## Conclusion

In this work, twenty-six electrical transformers were put through diagnostic and monitoring assessments. The proposed approach integrated DGA, transformer oil integrity analysis, visual inspections, and two BPNN algorithms to predict the LOL of the transformers through condition monitoring of the cellulose paper and the HI approach for prioritizing units for repair, maintenance, or replacement. The findings of the proposed diagnostic procedures were acquired and investigated, and a list of transformers was proposed for repair, maintenance, and continuous supervision.

Based on the diagnostic approach proposed in this study, the following conclusions were attained:

- The adoption of DGA and visual inspections on electrical transformers saves money when planning a maintenance schedule.
- The DGA data were used as input samples into the NN for diagnosis. The experimental findings demonstrated that the first BPNN algorithm could accurately forecast transformer DP. The first algorithm produced a correlation coefficient of 0.970 when the DP was predicted using the 2FAL measured in oil. It is essential to schedule transformer maintenance in advance to prevent failure from escalating.
- The results of the second BPNN algorithm were fed into the NN for assessment. The experimental results demonstrated that the second BPNN method was successful at forecasting transformer LOL. The second algorithm produced a correlation coefficient of 0.999 when the LOL was predicted using the 2FAL and DP output data obtained from the first algorithm.
- The experimental results demonstrated that the BPNN algorithms can overcome the constraints of other learning algorithms based on DGA due to the limited and disorganized dispersal of transformer oil data. The BPNN algorithm offers more effective generalization performance compared to the other learning algorithms.
- The HI findings revealed that no transformers had near-end-of-life or end-of-life materials as per the IEC 60599:2022 standard and CIGRE brochure.

Examining the condition of the cellulose paper insulation is crucial when thinking about a transformer maintenance schedule. In this instance, moving the transformer to a manufacturing station for rehabilitation would be a more economical maintenance strategy. All previous studies have demonstrated and verified that ${\text{MeOH}}$ appears to be an effective marker for detecting the start of cellulose paper degradation. ${\text{MeOH}}$ concentration increases in a “logarithmic’ trend (early diagnosis of deterioration), whereas 2FAL increases in an “exponential” trend (diagnosis when deterioration is extreme). When the cellulose insulation is exposed to low temperatures and the deterioration process is fully engaged, ${\text{MeOH}}$ content appears to stabilize. From this point on, 2FAL is considerably generated and becomes greater than ${\text{MeOH}}$. For future work, the authors will evaluate ${\text{MeOH}}$ and 2FAL concentrations using artificial intelligence algorithms. The conclusions about which data produces accurate results using the algorithms will be drawn from which data produces underfitting and overfitting during training.

## Data Availability

The data that support the findings of this study are available from the corresponding author upon reasonable request.
